# The expression of corticotropin-releasing hormone family peptides in premalignant and malignant vulvar lesions

**DOI:** 10.1007/s12094-023-03249-8

**Published:** 2023-06-29

**Authors:** Angelos Dimas, Anna Goussia, Alexandra Papoudou-Bai, Anastasia Politi, Minas Paschopoulos, Iordanis Navrozoglou, Antonis Makrigiannakis, Thomas Vrekoussis

**Affiliations:** 1 University of Ioannina, 45110 Ioannina, Greece; 2 Department of Pathology, Faculty of Medicine, School of Health Sciences, University of Ioannina, 45110 Ioannina, Greece; 3 Department of Pathology, University Hospital of Ioannina, 45110 Ioannina, Greece; 4 Department of Pathology, German Oncology Center, Limassol, Cyprus; 5 Department of Dermatology, Venereology, Andreas Syggros Hospital, National and Kapodistrian University of Athens, 16121 Athens, Greece; 6 Department of Obstetrics and Gynecology, Faculty of Medicine, School of Health Sciences, University of Ioannina, 45110 Ioannina, Greece; 7 Department of Obstetrics and Gynecology, School of Health Science, University of Crete, 71500 Iraklio, Greece

**Keywords:** CRH, Urocortin, CRHR1, CRHR2, FAS, FASL

## Abstract

**Objectives:**

To examine the relation of corticotropin-releasing hormone (CRH) family peptides with inflammatory processes and oncogenesis, emphasizing in vulvar inflammatory, premalignant and malignant lesions, as well as to investigate the possibility of lesion cells immunoescaping, utilizing FAS/FAS-L complex.

**Methods:**

Immunohistochemical expression of CRH, urocortin (UCN), FasL and their receptors CRHR1, CRHR2 and Fas was studied in vulvar tissue sections obtained from patients with histologically confirmed diagnosis of lichen, vulvar intraepithelial neoplasia (VIN) and vulvar squamous cell carcinoma (VSCC). The patient cohort was selected from a tertiary teaching Hospital in Greece, between 2005 and 2015. For each of the disease categories, immunohistochemical staining was evaluated and the results were statistically compared.

**Results:**

A progressive increase of the cytoplasmic immunohistochemical expression of CRH and UCN, from precancerous lesions to VSCC was observed. A similar increase was detected for Fas and FasL expression. Nuclear localization of UCN was demonstrated in both premalignant and VSCC lesions, with staining being significantly intensified in carcinomas, particularly in the less differentiated tumor areas or in the areas at invasive tumor front.

**Conclusions:**

Stress response system and CRH family peptides seem to have a role in inflammation maintenance and progression of vulvar premalignant lesions to malignancy. It seems that stress peptides may locally modulate the stroma through Fas/FasL upregulation, possibly contributing to vulvar cancer development.

## Introduction

During the last decades, we witnessed an increasing interest of the research community on the impact of the expression of hormones regulating response to stress—such as CRH family peptides and their corresponding receptors—as well as FAS/FASL apoptotic compound, on various physiological and pathological conditions [1–3]. However, the complexity of interactions of the numerous biological processes often leads to conflicting results among studies. From a biological point of view, the main evolutionary role of the human body’s response to stress is to promote survival through an alteration of the homeostatic status (allostasis). Despite its enormous significance in survival, the frequent need for neurobiological response to stressors or intense stress increases the risk of developing physical and mental health conditions [4].

Vulvar inflammatory diseases consist of a group of lesions of the vulva and the surrounding skin, that cause chronic or recurrent itching and pain in the area. The most common types of vulvar inflammatory diseases include lichen simplex chronicus, lichen sclerosus and lichen planus. Their appearance varies and overlaps; thus, biopsy constitutes the gold standard in ambiguous cases [5]. Nearly 75% of cases of vulvar cancer are related with vulvar inflammatory conditions, such as lichen sclerosus. Although lichen sclerosus per se is not considered a precancerous condition, it has been correlated with a higher risk of well-differentiated VSCC development [6].

Vulvar intraepithelial neoplasia (VIN) is an increasingly recognizable clinicopathological entity, causing significant physical and psychological morbidity. It is reported as a major cause of chronic itching or disturbance of the anatomy of the vaginal area, leading to discomfort and sexual disfunction [7]. There is a strong association between VIN and the development of VSCC; VIN lesions are often found alongside VSCC in 70–80% of the cases [7]. There are two distinct types of VIN that vary regarding the etiology, pathogenesis and clinical and pathological significance; VIN, HPV-associated is driven by HPV infection, and VIN, HPV-independent or differentiated VIN (dVIN) is related to inflammatory vulval conditions, regardless of HPV infection [8, 9]. Although the majority of VSCC are HPV-independent and dVIN is the best defined precursor lesion, the malignant potential of VIN–HPV associated should not be underestimated, with the risk of developing VSCC reaching 9–16%, if left untreated [7, 8, 10, 11].

On the other hand, vulvar cancer is generally considered an uncommon malignancy, representing only the 5% of all gynecological cancers and 1–2% of all cancers in women [12, 13]. Nevertheless, its incidence has risen from 2 to 21% in women under the age of fifty during the last two decades with the vast majority of them (90%) originating from squamous epithelial cells [14]. The etiopathogenesis of vulvar squamous cell carcinoma (VSCC) lies in two different mechanisms. The VSCCs, HPV-associated are related with HPV infection corresponding nearly 20–40% of all VSCCs, occur commonly in young females and histologically are moderately to poorly differentiated tumors, of basaloid or warty (condylomatous) morphology. VSCCs, HPV-independent are developed in the setting of lichen sclerosus or lichen planus or dVIN, affect usually older women and histologically are often well-differentiated keratinizing carcinomas and of verrucous morphology [8, 12, 15]. VSCC has a relatively good prognosis when diagnosed early, especially in patients with negative femoral lymph nodes at the time of first diagnosis. Unfortunately, one third of cases relapse [16]. Despite the extensive study of a plethora of biological markers in an effort to highlight correlations between their status and VSCC’s natural history, no definitive and useful in clinical management conclusions have been drawn to date.

CRH family peptides in adjunct with their related receptors CRHR1 and CRHR2 are major regulators of the human stress response system [17]. However, they have also been detected in various peripheral organs, such as skin, heart and lungs [18, 19], as well as the female reproductive system [20–23]. Their peripheral expression has also been reported in a large variety of human malignant cell lines, such as neuroblastoma, small-cell lung cancer and melanoma [24–28]. Nevertheless, their exact biological role in cancer remains uncertain and a subject of debate among researchers. To our knowledge, the role of CRH and CRH-like peptides on vulvar lesions—both precancerous and malignant—have not been extensively studied. The present study focuses on the investigation of the potential impact of stress regulating peptides, on pathogenesis and progression of chronic inflammatory diseases, premalignant and malignant lesions of the vulva.

FAS protein (CD95 or Apo-1) is a transmembrane protein consisting of 319 amino acids, belonging to the TNF and neurotrophic factors family peptides. It is highly expressed in various cells of the immune response system, including activated T and B lymphocytes, natural killer cells, monocytes and macrophages. The peptide that binds to FAS—or FASL (CD95L)—is also a transmembrane protein of the TNF family peptides and is expressed not only on the surface of activated T lymphocytes, but also by various cells that do not belong to the immune response system, such as Sertoli cells and the corneal epithelial cells. [1]. Both FAS and FASL seem to play an important role in regulating immune tolerance. The main biological effect of binding FASL to FAS is the induction of apoptosis of activated cells carrying the FAS protein on their cellular membrane [1].

In the present retrospective study, we investigated the immunohistochemical expression of CRH, UCN, FAS-L and of their receptors CRHR1, CRHR2 and FAS, on paraffin-embedded tissue samples from patients diagnosed with lichen (sclerosus or planus), VIN or VSCC. Given that inhibitors specific to the various CRH family peptides have already been developed, demonstrating peripheral expression of CRH and related peptides in the aforementioned lesions, would partly justify the experimental use of such inhibitors in the treatment of vulvar premalignant conditions and vulvar cancer. Additionally, the confirmation of immunoescape in vulvar cancer would strengthen the basis of a potential benefit after a local, iatrogenic immunomodulation.

## Materials and methods

### Study group

For the purposes of this study, formalin-fixed and paraffin-embedded tissue blocks of eighty seven (87) cases with histologically confirmed diagnosis of lichen plannus or sclerosus (*n* = 31 cases), VIN (*n* = 27 cases) and VSCC (*n* = 29 cases) were retrieved from the archives of the Pathology Department, University Hospital of Ioannina, Ioannina, Greece., The cohort included patients of all age groups diagnosed between 2005 and 2015. Hematoxylin–eosin (H&E)-stained sections from the tissue blocks were reviewed by two pathologists who recorded histological parameters in detail.

### Immunohistochemistry (IHC)

For immunohistochemical staining, 3 μm thick tissue sections were cut from representative tissue blocks and subsequently stained using IHC (Avidin–Biotin Complex) methodologies. The tissue slides were deparaffinized, rehydrated, enzyme digested and incubated with primary and secondary antibodies as per manufacturer’s protocols in each case. The IHC staining was performed using DAKO Autostainer LINK48 staining system for CRH, UCN and FAS detection and the automated VENTANA Benchmark XT staining system for CRHR1, CRHR2 και FAS-L detection. The following primary rabbit, polyclonal antibodies were used: anti-CRH/Pro CRH Picoband™ *(clone A00629; dilution 1:200; Boster Biological Technology U.S.A.)*, anti-Urocortin Antibody *(clone A01807; dilution 1/100; Boster Biological Technology U.S.A.)*, anti-FAS Antibody *(clone PB9252; dilution 1:200; Boster Biological Technology U.S.A.)*, as well as Rabbit CRHR1 Polyclonal Antibody *(clone MBS2552261; dilution 1:100; MyBioSource Inc., U.S.A.),* Anti- CRHR2 Antibody *(clone FNab01975, dilution 1:200; FineTest, Wuhan Fine Biotech Co., Ltd, CN)* and anti-FAS-L Antibody *(clone PA1576; dilution 1:300; Boster Biological Technology U.S.A.)*.

Negative controls were used by omitting the incubation process with the primary antibody in each case. Placental tissue was used as positive control for CRH and UCN immunostainings, lung carcinoma tissue for FAS immunostaining and thyroid, hepatocellular and colon cancer tissues were served as positive controls for CRHR1, CRHR2 and FAS-L immunostainings, respectively, according to the data sheet instructions.

Protein expression levels were evaluated by two independent pathologists using Nikon Eclipse 50i microscope. Immunostainings for CRH, CRHR1, CRHR2, FAS and FAS-L were cytoplasmic; whereas for UCN, both cytoplasmic and nuclear staining were detected. For immunohistochemical interpretation, a H-score staining score system was used, based on the following formula: H-score = [1 × (% cells 1 +) + 2 × (% cells 2 +) + 3 × (% cells 3 +)], with values from 0 to 300.

### Statistical analysis

The statistical evaluation of the results was carried out using the available from the University of Ioannina statistical software SPSS version 22 (IBM SPSS version 22, Armonk, NY, USA). For the statistical comparison of H-score results of each of immunohistochemical stainings (CRH, UCN, CRHR1, CRHR1, Fas and FasL), between the three disease categories (lichen, VIN, VSCC), the non-parametric Kruskall–Wallis *H* test was used. In those cases where the test led to the rejection of the null hypothesis (no statistical significance in the H-score variation of immunoassay distributions between the three disease categories), a Mann–Whitney *U* test was performed. In all cases, *p* < 0.05 was used as the limit of statistical significance. Finally, especially for UCN antibody staining, nuclear staining in various disease categories was evaluated as well.

## Results

### Immunohistochemical expression of CRH

**Fig. 1:**
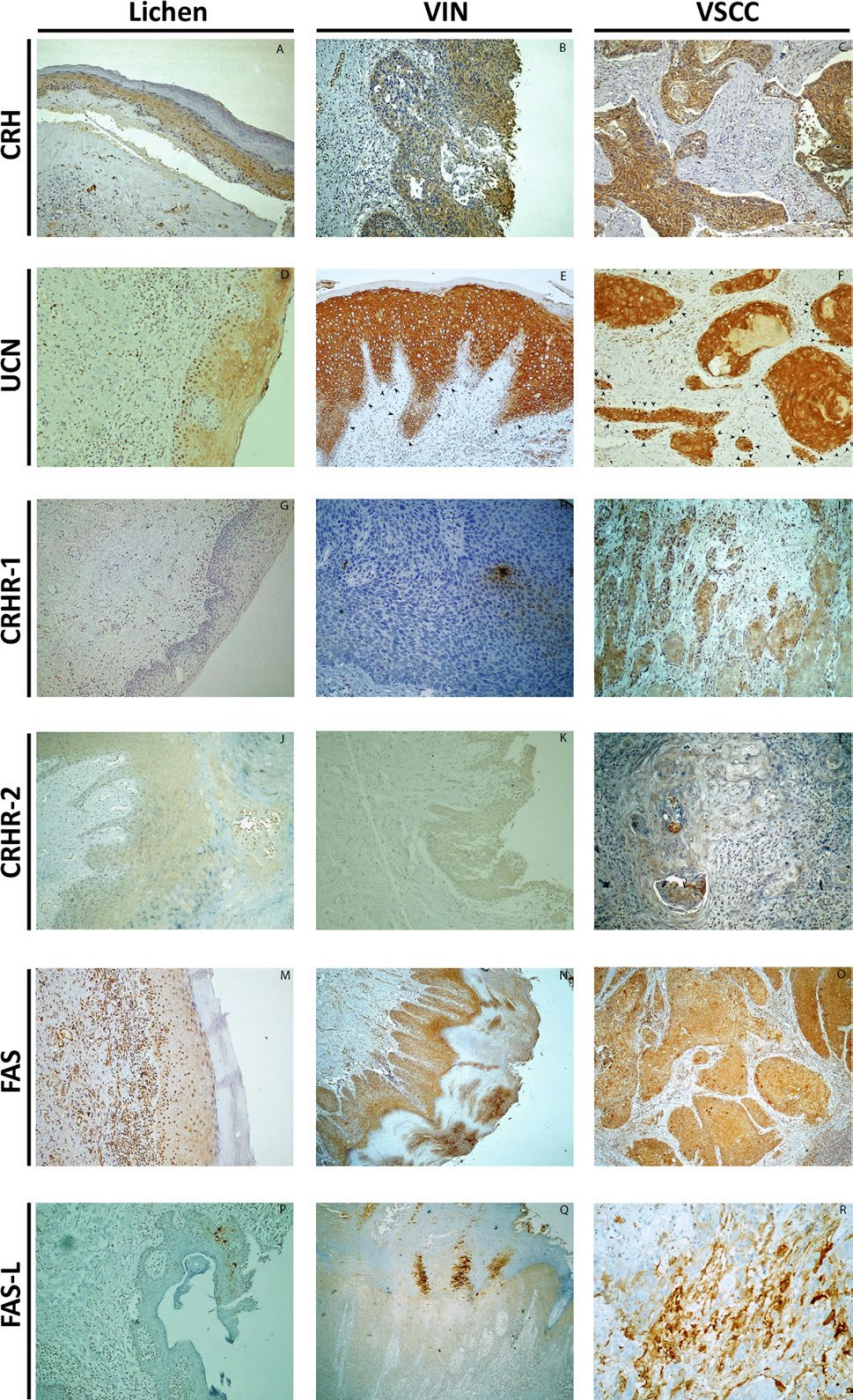
Representative captions after immunohistochemical staining of vulvar tissues according to research protocol. Arrows in VSCC depict nuclear positivity for UCN in malignant cells of the invasive front. (Captions D, H and R are provided in ABC X200, N, O and Q in ABC × 40, while the rest are in ABC × 100)

Immunohistochemical expression of CRH was evaluated in 26 cases of lichen, 26 cases of VIN and 29 cases of VSCC. Representative captions of all immunohistochemical stains conducted are depicted in Fig. 1. Staining was diffuse cytoplasmic, weak in intensity in lichen cases, and more intense in VIN and VSCC cases. The mean H-score was 36.2, 89.4 and 127.4 in lichen, VIN and VSCC, respectively. Statistical analysis showed that CRH expression was significantly higher in VSCC compared to VIN (*U* = 175, *Z* =  − 3.4, *p* = 0.001 two tailed) and lichen (*U* = 30, Z =  − 5.9, *p* < 0.001 two tailed). Similarly, CRH expression was higher in VIN cases, compared to lichen cases (*U* = 77.5, *Z* =  − 4.8, *p* < 0.001 two tailed) (Table 1).

**Table 1:** Mann–Whitney *U* Test results of staining against CRH between lichen, VIN and VSCC samples

CRH	Mann–Whitney *U*	*Z*	ASYMP. SIG. (2-tailed)
LICHEN—VIN	77.5	− 4.8	< 0.001
VIN—VSCC	1	− 3.4	0.001
LICHEN—VSCC	30	− 5.9	< 0.001

### Immunohistochemical expression of UCN

Thirty-one (31) cases of lichen, 27 cases of VIN and 29 cases of VSCC were studied in respect to UCN expression. UCN expression was evaluated and compared separately in the cytoplasm and the nucleus in the various histological samples. Although UCN cytoplasmic expression was detected in all three disease categories, statistical analysis failed to detect any statistical difference among them (Chi square = 0.402, *p* = 0.818, df = 2). The mean H-score was 162.4, 149.6 and 153.8 in lichen, VIN and VSCC samples, respectively.

Interestingly, the analysis detected significant differences considering the nuclear expression of UCN among the three disease categories. In VIN samples (mean H-score = 20.6), nuclear expression was found higher compared to lichen samples (*U* = 293, *Z* =  − 2.4, *p* = 0.015 two tailed, mean H-score = 6.5). Similarly, nuclear UCN expression was higher in VSCC cases (mean H-score = 26), compared to both VIN (*U* = 274, *Z* =  − 2, *p* = 0.047 two tailed) and lichen cases (*U* = 159.5, *Z* =  − 4.7, *p* < 0.001 two tailed) (Table 2).

**Table 2:** Mann–Whitney *U* Test results of nuclear staining against UCN between lichen, VIN and VSCC samples

nUCN	Mann–Whitney *U*	Z	ASYMP. SIG. (2-tailed)
LICHEN—VIN	293	− 2.4	0.015
VIN—VSCC	274	− 2	0.047
LICHEN—VSCC	159.5	− 4.7	< 0.001

### Immunohistochemical expression of CRHR1

Expression of the receptor CRHR1 was studied in 31, 27 and 29 cases of lichen, VIN and VSCC, respectively. Immunohistochemical staining was diffuse cytoplasmic. Although no differences were found between VIN (mean H-score of 0.8) and lichen cases (*U* = 387.5, *Z* =  − 1.5, *p* = 0.126 two tailed, mean H-score of 0), CRHR1 expression in VSCC samples (mean H-score of 6.5) was significantly higher compared to both VIN (*U* = 283, *Z* =  − 2.5, *p* = 0.013 two tailed) and lichen samples (*U* = 294.5, *Z* =  − 3.5, *p* < 0.001 two tailed) (Table 3).

**Table 3:** Mann–Whitney *U* Test results of staining against CRHR1 between lichen, VIN and VSCC samples

CRHR1	Mann–Whitney *U*	*Z*	ASYMP. SIG. (2-tailed)
LICHEN—VIN	387.5	− 1.5	0.126
VIN—VSCC	283	− 2.5	0.013
LICHEN—VSCC	294.5	− 3.5	< 0.001

### Immunohistochemical expression of CRHR2

CRHR2 receptor expression was evaluated in 31 lichen, 26 VIN and 29 VSCC samples. Immunostaining was absent to weak in all samples—mean H-score of 3.5, 5.8 and 4.1 in lichen, VIN and VSCC, respectively. Statistical analysis found no significant differences between the three disease categories (Chi square = 4.5, *p* = 0.108, df = 2).

### Immunohistochemical expression of FAS

Twenty-six (26) lichen, 25 VIN and 27 VSCC samples were stained for FAS protein and a diffuse cytoplasmic pattern was observed. Kruskal–Wallis *H* test detected significant variability of FAS expression between sample categories; so, Mann–Whitney *U* tests were performed. The expression was significantly higher in VIN (mean H-score 69.2), compared to lichen samples (*U* = 116.5, *Z* =  − 4, *p* < 0.001 two tailed, mean H-score 4.6). Additionally, FAS expression in VSCC cases (mean H-score 110) was more prominent when compared to VIN (*U* = 211.5, *Z* =  − 2.3, *p* = 0.021 two tailed) and lichen cases (*U* = 83.5, *Z* =  − 4.8, *p* < 0.001 two tailed) (Table 4).

**Table 4:** Mann–Whitney *U* Test results of staining against FAS between lichen, VIN and VSCC samples

FAS	Mann–Whitney *U*	*Z*	ASYMP. SIG. (2-tailed)
LICHEN—VIN	116.5	− 4	< 0.001
VIN—VSCC	211.5	− 2.3	0.021
LICHEN—VSCC	83.5	− 4.8	< 0.001

### Immunohistochemical expression of FAS-L

Immunohistochemical staining for FAS-L was performed in 31 lichen, 26 VIN and 29 VSCC samples, respectively. The staining was cytoplasmic and weak in all three disease categories. However, statistical analysis showed a significantly higher FAS-L expression in VIN (mean H-score 10.4), compared to lichen lesions (mean H-score 1.4) (*U* = 173.5, *Z* =  − 3.8, *p* < 0.001 two tailed). Additionally, expression in VSCC (mean H-score 28.4) was notably higher as compared to that of VIN (*U* = 123, *Z* =  − 4.3, *p* < 0.001 two tailed) and lichen samples (*U* = 12, *Z* =  − 6.5, *p* < 0.001 two tailed) (Table 5).

**Table 5:** Mann–Whitney *U* Test results of staining against FASL between lichen, VIN and VSCC samples

FASL	Mann–Whitney *U*	*Z*	ASYMP. SIG. (2-tailed)
LICHEN—VIN	173.5	− 3.8	< 0.001
VIN—VSCC	123	− 4.3	< 0.001
LICHEN—VSCC	12	− 6.5	< 0.001

## Discussion

Both CRH and UCN are important molecules involved in stress regulation. It has been shown that both neuropeptides are expressed in various tissues mediating and regulating human body’s local inflammatory response and reaction to stressors [3].

It is already known that various normal tissues of the female reproductive system, such as endometrium, myometrium and ovary, express CRH [21, 23, 29]. Furthermore, the expression of CRH has been reported in endometrial [21, 30], ovarian [2] and cervical [3] cancer. To our knowledge, the present study is the first one that investigated the immunohistochemical expression of the CRH peptides in vulvar premalignant and malignant lesions.

CRH expression was found to be gradually increasing from lichen to vulvar carcinoma with the latter being found to maintain high levels of CRH. In line with the current results, it was previously reported that the expression of CRH was upregulated in skin cancer, compared to cutaneous premalignant lesions [31]. The importance of this CRH augmentation at the transition from precancer to cancer has been also considered as an inducer of angiogenesis and tumor cell migration [32, 33]. Knowing that the CRH family is featuring local inflammation, it could be hypothesized that the initial inflammation of lichen sclerosus could favor the gradual development of VIN and cancer thereafter. Inflammation as part of cancer progression would further up-regulate CRH expression.

The role of CRH in establishing immune modulation was initially demonstrated in human reproduction. It was shown that maternal–fetal tolerance was mediated by the CRH-induced FasL expression which in turn triggered Fas-expressing T-cell apoptosis. This mechanism was later shown to contribute to tumor immune escape in both ovarian and cervical cancer. Concerning ovarian cancer, *Minas *et al*.* reported a statistically significant increase in CRH and FASL expression levels in the advanced stage of the disease [2]. Moreover, the authors found that incubation of ovarian carcinoma cells with CRH resulted in FASL upregulation, through activation of CRHR1 receptors, ultimately inducing increased apoptosis of activated T lymphocytes. In the present study, FASL expression was significantly elevated in VIN compared to lichen and in VSCC compared to VIN cases. This observed progressive increase is in accordance with the previously reported finding of FASL being overexpressed in cervical cancer cells and in its precancerous lesions (CIN) constituting a biological modification occurring in the early stages of carcinogenesis [34]. In line with the above-cited study, FASL upregulation could be hypothesized to be the effect of local CRH to vulvar epithelium favoring the local scheme of immune escape [2].

The biological role of UCN consists of both pre-inflammatory and anti-inflammatory effects [35]. In fact, these effects are mediated by CRHR1 and CRHR2 receptors. This dual role of UCN is likely to be determined by the different phases of inflammation and by local concentrations of the peptide, in addition to the type of target tissues and cells [35]. In our study, strong immunohistochemical cytoplasmic staining against UCN was observed in both lichen and VIN samples, possibly highlighting the potential contribution of this peptide in intense inflammatory response. A highly positive staining was also observed in VSCC samples. However, no significant difference was found between the three entities under study. On the contrary, nuclear UCN staining was progressively positive. Specifically, regarding VSCC cases, it was observed that in tumor cells found to the invasive front or in the less differentiated tumor areas, nuclear staining was more intense, with the cytoplasmic one appearing weak or even absent. However, in well-differentiated tumor areas, cytoplasmic staining was more prominent. In precancerous lesions, nuclear UCN staining was detected only in cells of the basal layer. Currently, the evidence upon UCN expression is rather weak. UCN has been shown to be upregulated in endometriosis [36], down-regulated in endometrial carcinoma [37]; whereas, it is reported as present in prostate, renal and gastric adenocarcinoma [38–40]. However, the increased nuclear intensity especially in less differentiated tumors has been reported before with UCN nuclear positivity being significantly profound in cases of human astrocytomas and gliomas, compared to reactive gliosis [41]. The correlation between UCN nuclear positivity and tumor differentiation warrants further investigation.

CRHR1 expression in lichen and VIN samples was practically undetectable apart from a weak staining pattern observed in some VSCC samples. Nevertheless, this weak expression proved to be of statistical significance when comparing VSCC samples with both VIN and lichen lesions. In our study, expression of CRH appeared to be progressively increased between lichen, VIN and VSCC samples. One would expect CRH’s specific receptor—CRHR1—to follow the same pattern. However, it could be suggested that this minimal CRHR1 upregulation in VSCC could be the result of CRH expression regulation. There are no studies in literature comparing CRHR1 expression in carcinomas and premalignant lesions so far. Of note is the report that chronic CRH stimulation of endometrial and myometrial, as well as pituitary cells, can act as a negative-feedback stimulus, ultimately reducing CRHR1 expression on target cells’ membrane [42]. On the other hand, other studies suggest that expression of CRHR1 receptor in endometrial cancer cells (Ishikawa cells), is not affected by its binder [43].

To the same direction, immunohistochemical staining against CRHR2 was weak in all three diseases studied, with no significant variability among them. Perhaps, this could be attributable to a CRH/UCN negative feedback. CRHR2 downregulation in vulvar cancerous and precancerous lesions is in accordance with the previous studies reporting that lack of CRHR2 receptor expression in colon cancer seems to (a) promote tumor growth and proliferation, maintaining a chronic inflammatory environment through continued activation of STAT3 [44] and (b) contribute to colon cancer cells’ resistance to apoptosis through FAS/FASL activation [45]. Interestingly, CRHR2 downregulation has also been demonstrated in renal cell carcinoma cases highlighting a disruption of an anti-angiogenic pathway [39].

It is known that, most—if not all—types of cancers are resistant to cellular apoptosis through FAS/FASL, even when overexpressing FAS receptor on their cell membrane [46]. In cases analyzed herein, a statistically significant increase in FAS expression was detected in VSCC, in comparison to VIN and lichen lesions. These results seem to contradict the theory of inhibition of apoptosis through activation of FAS/FASL, as this would require a reduction in FAS expression levels in malignant cells, as possibly it happens in cervical cancer [47]. Malignant cells also utilize other mechanisms of immunoescape, except from regulation of these receptors. Inhibition of FAS/FASL apoptotic pathway may also take place at the level of death-inducing signaling complex (DISC) formation, through cFLIP upregulation. Additionally, FAS receptor is associated with several other, non-apoptotic biological processes. It has been reported that FAS receptor is associated with cell proliferation of various cell types, such as hepatocytes, T-cells and nervous cells. Moreover, FAS stimulation is in vitro associated with cellular mobility and metastatic potential increase of malignant cell lines, resistant to apoptosis. In general, it is widely accepted that once malignant neoplastic cells develop resistance to apoptosis via FAS/FASL, further stimulation of FAS is considered oncogenic [46].

As several studies report, it is possible that initially, malignant cells downregulate the expression of FAS receptor, in an attempt to escape the host’s immune system response mechanisms, avoiding apoptosis. The majority of studies, however, highlight the possible link between cancer and gradual FAS—and sometimes also FASL—overexpression. Specifically, in gynecological malignancies studied to date, expression of FAS and especially FASL seems to be associated with tumor growth promotion and metastasis, as well as with a worse overall patients’ prognosis [46–50]. To our knowledge, this is the first study in an effort to detect FAS receptor expression in vulvar malignancies, using immunohistochemistry. Although the use of FASL as a target molecule for anticancer therapy is not an option due to its hepatocellular apoptosis induction, FAS receptor may pose a possible future anticancer therapy target in vulvar malignancies, once its biological function is fully elucidated.

A potential weakness of our study is the fact that FAS/FASL was not analyzed in the stroma in terms of correlating it with specific immune cell compartments (like locally existing T cells and macrophages). Such an approach although appealing, would require double immunohistochemistry staining that is rather difficult to be performed in formalin-fixed paraffin-embedded tissues. A future approach, applying both tissue immune-staining and cell co-culture experiments could produce the evidence to further strengthen our thesis.

## Conclusions

In conclusion, herein, we demonstrated a possible role of CRH family peptides in vulvar cancer and its premalignant lesions. It was clearly shown that CRH expression is gradually increased during the transition from lichen sclerosus to VIN and vulvar cancer, thus implying a possible role for CRH and its downstream pathways in local modulations towards an anti-apoptotic cellular scheme. Whether these hormones play a causal role in carcinogenesis or not remains to be elucidated. Our results demonstrated an upregulation of FASL by malignant neoplastic cells, a fact that could be anticipated in the frame of local immune suppression previously reported in several other cancers. Interestingly, apart from FASL, FAS expression was also upregulated in VSCC. Of note, FAS overexpression in various other malignancies has already been associated with tumor growth and development promotion and with a less favorable prognosis. Our present study should be considered as a preliminary effort to outline the role of CRH and UCN pathways in vulvar cancer pathophysiology. The results presented herein could support the design and study of novel anti-CRH receptor inhibitors as pharmaceutical intervention in vulvar cancer prevention. Undoubtedly, further studies are needed to verify our findings to formulate a working hypothesis of CRH-based interventions in vulvar precancer and cancer.

## Data Availability

The data that support the findings of this study are available from the corresponding author upon reasonable request.
